# Combined Double-Coil and Handheld rPMS in Low Back Pain: An Observational Case Series Based on Routine Clinical Practice

**DOI:** 10.3390/life16040594

**Published:** 2026-04-02

**Authors:** Vincenzo Di Modica, Giuseppe J. Sciarrone, Miloš Barna

**Affiliations:** 1 Rehabilitation Center, BioSalus, 87036 Surdo, Italy; 2 Spine and Robotic Surgery Centre, Humanitas Pio X, 20090 Milan, Italy; 3 Rehabilitation Medicine Clinic, Barna Medical, 130 00 Prague, Czech Republic

**Keywords:** low back pain, electromagnetic fields, lumbosacral region, buttocks

## Abstract

Despite the high prevalence of low back pain (LBP), evidence supporting the clinical effects of repetitive peripheral magnetic stimulation (rPMS) remains limited. A combined double-coil and handheld rPMS approach may enhance the therapeutic potential of this technology. This observational case series analyzed prospectively collected routine clinical data from 37 patients treated with a combined protocol of static double-coil lumbar rPMS and dynamic handheld lower-limb rPMS. Pain intensity, disability, and quality of life were assessed at baseline, post-treatment, and 1-month follow-up. Statistical analyses were complemented by an evaluation of clinical relevance using established minimal clinically important difference (MCID) thresholds. Significant improvements were observed across all outcomes. Pain decreased by 62.5% post-treatment and by 87.5% at follow-up, while disability was reduced by 86.8% and 92.1%, respectively. Quality of life scores approximately doubled. High MCID responder rates and consistent within-group changes were observed; however, given the single-arm design without a control group, these findings should be interpreted as exploratory and hypothesis-generating rather than confirmatory evidence of treatment effectiveness. This combined rPMS approach was feasible and well tolerated in routine clinical practice and was associated with clinically meaningful within-group improvements in pain, function, and quality of life. Further controlled studies are warranted.

## 1. Introduction

Low back pain (LBP) is one of the most common musculoskeletal conditions worldwide, affecting up to 85% of the population during their lifetime. It is also a leading cause of work-related disability and reduced quality of life [1]. Due to these factors, the management of LBP has significant socioeconomic implications and, for example, in the United States it accounts for the third highest national health expenditure [2]. In most cases, LBP is non-specific and multifactorial, with contributing factors including lumbar disc degeneration, soft tissue strain, impaired movement patterns, and postural dysfunction [3,4].

Even in cases of persistent or recurrent pain, LBP is not necessarily accompanied by abnormal MRI findings [5]. Symptoms often have a negative impact on daily activities, particularly among individuals in older working-age groups. The burden of disability caused by LBP is greatest among people aged 45–49 years, reflecting its substantial effect on quality of life and productivity during the most active period of life [2]. Moreover, the resulting disability can significantly influence mental health, leading to elevated levels of anxiety and depression, as well as reduced overall well-being [6].

Current treatment strategies for LBP vary widely, ranging from pharmacological management and physiotherapy to more invasive approaches depending on the examination findings. In Europe, physiotherapy remains the predominant treatment method (88%), followed by pharmacological therapy (53%) and surgical interventions (26%) [4].

In addition to conventional treatment methods for LBP, repetitive peripheral magnetic stimulation (rPMS) is becoming increasingly popular. So far, its clinical use has been mainly documented in the treatment of spasticity and motor function in neurological patients after stroke or in those with other neurological disorders such as cerebral palsy or multiple sclerosis [7,8]. In these populations, rPMS primarily targets impaired central motor pathways and neuroplastic mechanisms. More recently, this technique has gained attention in the management of musculoskeletal conditions for pain reduction, improvement of muscle strength, and enhancement of functional performance in daily activities [9]. In the treatment of LBP, however, the evidence for rPMS remains relatively limited, although several studies have demonstrated its effects on pain, disability, postural control, and psychological well-being in patients with LBP [3,10,11]. Importantly, LBP represents a predominantly multifactorial musculoskeletal condition rather than a primary central neurological disorder, and therefore the mechanisms described in neurological populations cannot be directly extrapolated. The proposed mechanism of action in musculoskeletal populations involves neuromuscular activation of superficial muscles, enhancement of local blood flow and proprioceptive input, as well as potential modulation of cortical motor areas involved in postural control and pain perception, although the relative contribution of peripheral and central mechanisms in LBP remains incompletely understood [3,8,11].

Although rPMS is a well-established therapeutic modality, the underlying technology has seen limited innovation [12]. In physiotherapy practice, two types of rPMS applicators are commonly used—the single-coil applicator and the figure-8 applicator, which consists of two coils placed side by side without the possibility of mutual tilting to anatomically conform to the treated area [12,13]. Both applicator types are typically used in a static position, without the option for dynamic adjustment during therapy. While dynamic coil positioning has been explored in experimental transcranial magnetic stimulation (TMS) systems using robotic or electronically controlled arrays [14,15], such approaches have not yet become part of standard therapeutic procedures in physiotherapy. Likewise, the clinical effects of a double-coil applicator that allows for mutual tilting of the coils—enabling better anatomical conformity—have only recently begun to be explored in physiotherapy settings. To date, only a limited number of pilot clinical studies have evaluated the effects of double-coil rPMS, reporting positive outcomes in disorders of upper and lower extremity joints, including knee osteoarthritis [16,17]. However, no clinical studies have yet investigated the application of a combined static, tiltable double-coil applicator together with a dynamic handheld applicator in patients with low back pain.

Broader-field stimulation at lower intensity may be preferable for LBP, as rPMS acts through distributed neuromuscular and cortical pathways rather than focal excitation alone [3,18]. A recent study comparing single- and double-coil rPMS configurations demonstrated that while single coils produce higher stimulation intensity and are more strongly perceived in the lower back region, double-coil applicators cover nearly twice the area and offer improved patient comfort [19]. Although double coils deliver lower peak intensity, their broader spatial coverage—especially when combined with hand-held dynamic stimulation—may theoretically facilitate more extensive activation of myofascial structures across the lumbopelvic and posterior thigh regions. This expanded stimulation area may enhance neuromuscular modulation and therapeutic outcomes, particularly for indications such as muscle relaxation and postural reconditioning in LBP [20,21]. However, the clinical implications of such combined stimulation remain insufficiently explored.

Moreover, LBP is not always isolated to the lumbar region. Up to 68% of musculoskeletal patients experience pain affecting multiple regions, most commonly involving both the lumbar and gluteal or lower-limb areas [22,23]. In some cases, dysfunction of the gluteal muscles may also contribute to LBP, primarily due to muscle weakness, altered activation patterns, and increased fatigability [23,24,25]. Impaired load transfer from the gluteal region to the lumbar spine may represent a contributing factor or even a source of LBP [26]. A combined therapeutic approach that not only stimulates the lumbar area but also dynamically targets the gluteal and posterior thigh muscles may therefore better address the myofascial and neuromuscular components of LBP.

Given the limited evidence on combined rPMS protocols targeting both lumbar and lower-limb regions, and the absence of clinical data on tiltable double-coil applicators in musculoskeletal populations, exploratory observational data from routine clinical practice may provide initial insight into the feasibility and potential clinical impact of such an approach.

The aim of this observational case series, based on prospectively collected routine clinical data, was to evaluate the feasibility and preliminary clinical effects of a novel rPMS protocol, rather than to isolate the effects of its individual components.

This approach combines static double-coil lumbar stimulation with dynamic handheld gluteal and posterior thigh stimulation in patients with LBP. This specific combined approach has not been previously described in the literature in patients with LBP. Its clinical potential will be interpreted in the context of existing evidence on standard, statically applied rPMS.

## 2. Materials and Methods

### 2.1. Study Design

This observational case series was based on prospectively collected data obtained during routine clinical practice to evaluate the feasibility and preliminary effects of a novel rPMS protocol in patients with LBP. The intervention was introduced as part of standard rehabilitation care during a period of clinical implementation of a newly available double-coil rPMS device, and no predefined research hypothesis or trial protocol was established at the time of treatment initiation. The study included only an experimental group and was designed to explore clinical outcomes and patient tolerability under real-world rehabilitation conditions.

Data were analyzed from patients who had undergone the combined rPMS protocol, met all inclusion and exclusion criteria, provided informed consent, and had complete outcome records at all three checkpoints. Clinical outcomes (VAS, ODI, PGIC, SF-12) were systematically recorded at predefined time points as part of internal quality monitoring procedures. The 1-month follow-up assessment was conducted through structured telephone or email communication, during which patients submitted scanned copies of the completed questionnaires identical to those used during in-person assessments. Given the observational design without a control group, the analysis was intended to describe within-group changes rather than to establish causal treatment effects.

The study protocol for secondary analysis of anonymized clinical data was reviewed and approved retrospectively by the local ethics committee Comitato Etico Territoriale Regione Calabria (319/2025, 29 December 2025). The ethics committee evaluated and approved the secondary scientific analysis of anonymized routine clinical data. All participants were informed in detail about the treatment protocol, potential risks, and the possibility of anonymized data publication. Written informed consent was obtained from each participant prior to the intervention as part of standard clinical documentation, including permission for anonymized use of clinical data for research and publication purposes. The study was conducted in accordance with the principles outlined in the Declaration of Helsinki.

In addition to the clinical analysis, the study incorporated a structured literature review to provide narrative contextualization of the findings rather than formal comparative evaluation.

### 2.2. Inclusion/Exclusion Criteria

Patients were identified from the database of a private outpatient physiotherapy clinic specializing in the treatment of musculoskeletal disorders. Eligibility was limited to adult patients with acute or chronic LBP, defined as pain located between the lower costal margin and the gluteal folds, with or without associated functional limitation. All participants were required to be capable of providing informed consent and complying with the treatment protocol.

Exclusion criteria included pregnancy; the presence of any implanted electronic or metallic devices (e.g., pacemakers, defibrillators, neurostimulators, or metal-containing intrauterine devices); active drug infusion systems; a history of seizures or epilepsy; diagnosed malignancy; severe cardiovascular, pulmonary, or renal disease; systemic infections, febrile states, or open wounds in the treatment area; or any neurological or musculoskeletal condition unrelated to LBP that could interfere with outcome evaluation.

Only patients who fulfilled all eligibility criteria, signed informed consent, and completed all checkpoints—including the 1-month follow-up—were included in the final analysis. This selection approach reflects a complete-case analysis of patients with fully documented routine clinical data.

### 2.3. Intervention

The intervention consisted of a combination of static double-coil and dynamic handheld rPMS applied to the lumbar and lower-limb regions. The treatment was delivered using the Super Inductive System Duo (BTL Industries, Ltd., Prague, Czech Republic).

A dual-coil applicator was positioned over the lumbar spine, targeting the paraspinal region at the level of the clinically relevant lumbar segment identified during examination. The stimulation protocol followed a predefined manufacturer default sequence designed for chronic pain conditions, with a total duration of 10 min and consisting of six consecutive sections. The protocol incorporated amplitude-modulated and frequency-modulated phases, with frequencies ranging between approximately 1 and 50 Hz depending on the program section. Section 1 consisted of an alternating frequency phase (50–5 Hz) serving as an initial activation and adaptation phase. Section 2 applied low-frequency stimulation (1 Hz), forming a transitional phase before the main analgesic modulation. Section 3 and Section 4 applied low-frequency alternating modulation (1–10 Hz) aimed at analgesic neuromodulation. Section 5 included higher-frequency stimulation (approximately 50 Hz) intended to activate the micromuscular pump and support local circulation. The final section (1 Hz) served as a short calming phase. Amplitude was predefined within the device program (0–100% of system output), with gradual rise and fall times automatically controlled by the system.

Simultaneously, a hand-held applicator was used to deliver dynamic rPMS manually over the gluteal and posterior thigh regions, particularly in patients presenting with referred lower-limb pain. The protocol comprised sequential phases: (1) manual analgesic action on the myofascial structures of the lumbar and lower-limb regions, (2) improvement of local trophism and circulation, (3) analgesic neuromodulation of the affected myofascial areas, and (4) progressive activation of the lumbar and lower-limb musculature as symptoms subsided. The anatomical regions stimulated and the sequence of application were standardized, while intensity was individualized according to patient tolerance.

In both static and dynamic applications, output intensity was initially set below the sensory threshold and gradually increased according to clinical improvement to achieve neuromotor activation. While frequency parameters, pulse shape, and amplitude modulation were predefined within the selected program, output intensity was individualized and adjusted throughout the session based on the patient’s tolerance and real-time response.

**Figure 1:**
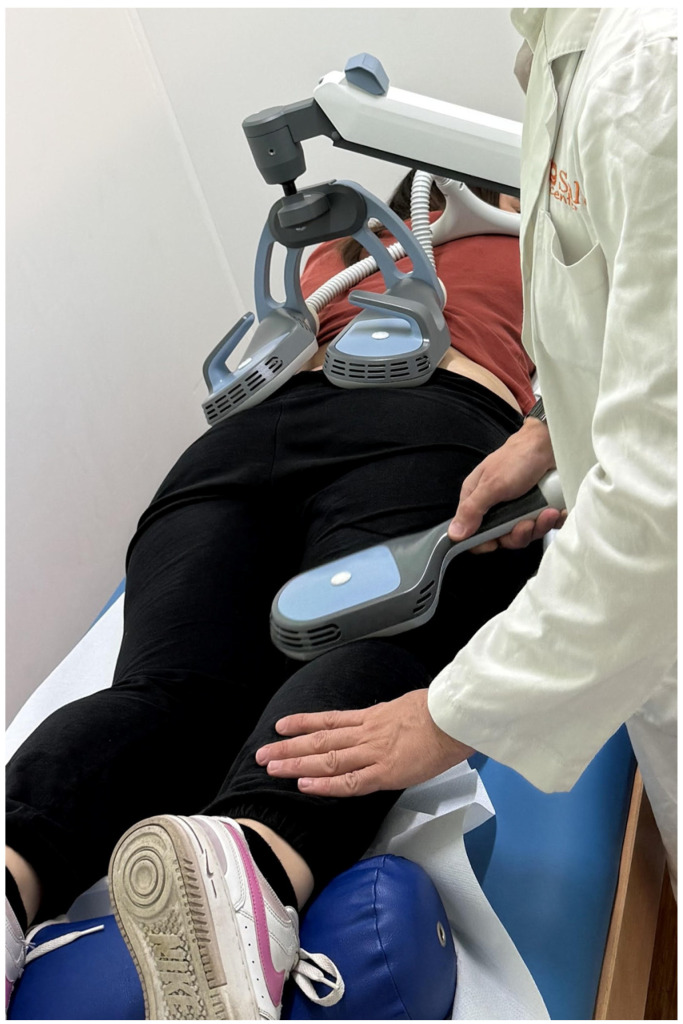
Therapy setup of double-coil rPMS combined with dynamic handheld approach.

Participants completed a total of nine sessions (three per week over three consecutive weeks). An example of the therapy setup is shown in Figure 1.

### 2.4. Outcome Measures

Clinical outcomes were assessed using a set of validated self-reported instruments commonly employed in LBP research. All measures were collected at three time points: baseline, post-treatment, and 1-month follow-up.

The following instruments were used:

• Visual Analogue Scale (VAS) for pain intensity: A 10 cm horizontal line ranging from “no pain” to “worst imaginable pain.” Scores range from 0 to 10, with higher scores indicating greater pain intensity [27].
• Oswestry Disability Index (ODI): A questionnaire assessing functional disability associated with LBP. The ODI includes ten items, each scored from 0 to 5, with a total score expressed as a percentage (0–100%). Higher scores indicate more severe disability, with 0% representing no disability and 100% indicating maximum limitation [28].
• Patient Global Impression of Change (PGIC): A multidomain PGIC format, as described by Scott et al., was used to assess patient-perceived change following treatment. In contrast to the traditional single-item PGIC, this format applies the standard 7-point PGIC response scale (1 = very much improved to 7 = very much worse) to six predefined domains: overall condition, physical activities, social activities, work-related activities (including household tasks), mood, and pain. A composite PGIC score was calculated as the mean of the six domain scores for descriptive purposes [29].
• Short Form-12 Health Survey (SF-12): A health-related quality of life instrument that produces two component scores: Physical Component Summary (PCS) and Mental Component Summary (MCS). Higher scores reflect better self-reported physical and mental health [30].

Minimal clinically important difference (MCID) thresholds were defined according to published literature: ≥2 points for VAS, ≥10 points (or ≥20%) for ODI, and ≥3.4 points for SF-12 PCS and 4 points for SF-12 MCS [31,32,33]. Clinical significance was further expressed as the MCID responder rate, representing the percentage of patients exceeding these thresholds. Given the observational design without a control group, MCID analyses were interpreted as descriptive indicators of within-group change rather than evidence of comparative treatment efficacy.

The 1-month follow-up assessment was conducted remotely via structured telephone or email contact. Patients received the same standardized questionnaires used during in-person assessments and were instructed to complete them independently without assistance. No therapeutic guidance or feedback was provided during follow-up communication. Completed questionnaires were returned in written form. This standardized procedure was intended to ensure consistency with baseline and post-treatment assessments. However, given the self-reported and remote nature of follow-up data collection, the possibility of response bias cannot be fully excluded.

### 2.5. Literature Search Strategy

A structured literature search was conducted to identify clinical studies evaluating rPMS in the treatment of LBP. The aim was to contextualize the present observational case series within existing evidence and to provide narrative comparison of outcome patterns rather than formal methodological comparison with previously published protocols.

The search was performed using the Consensus academic search engine, which aggregates peer-reviewed publications from PubMed, CrossRef, Semantic Scholar, and other biomedical databases. Three search strategies were used:

• “peripheral magnetic stimulation” AND “lumbar spine”;
• (“repetitive magnetic stimulation” OR “rPMS”) AND (“low back pain” OR “lumbar pain”);
• “repetitive peripheral magnetic stimulation” AND “low back pain”.

Only studies published in English were included. Studies had to:

• Be primarily focused on LBP;
• Investigate rPMS as a standalone intervention (i.e., not combined with TENS, rTMS, injections, or other modalities);
• Report at least one of the following outcomes: VAS/NPRS, ODI, or SF-12/SF-36.

Studies were excluded if they:

• Focused primarily on radicular syndromes or sciatica without axial LBP as the primary symptom;
• Combined rPMS with other active interventions in a way that would confound its isolated effect;
• did not provide relevant outcome data or were only published as abstracts, technical notes, or in non-peer-reviewed formats.

The study identification and selection process is presented using a PRISMA flow diagram to enhance transparency and reproducibility of the search strategy. However, the review was not conducted as a formal systematic review or meta-analysis, but rather as a structured narrative search intended to contextualize the present findings within the available clinical evidence.

### 2.6. Data Analysis

All statistical analyses were performed using GraphPad Prism version 10 for Windows (GraphPad Software, Inc., San Diego, CA, USA). Normality of data distribution was assessed using the Shapiro–Wilk test. Outcome measures did not meet the assumption of normality and therefore were analyzed using the non-parametric Wilcoxon Signed Rank Test and reported as median and interquartile range (IQR). Treatment-induced changes were expressed as median change (Δ) (IQR) with Hodges-Lehmann 95% confidence intervals (CI), accompanied by rank-biserial effect sizes (r) and MCID responder rates. The rank-biserial coefficient was used as it represents the natural effect size associated with the Wilcoxon Signed Rank Test and is appropriate for paired, non-normally distributed clinical data. Statistical significance was determined at a threshold of *p* < 0.05. Given the single-arm observational design without a control group, statistical analyses were interpreted as descriptive assessments of within-group change rather than evidence of comparative treatment efficacy.

Sample size was determined by the availability of complete clinical records meeting the inclusion and exclusion criteria. As this study represents an observational case series based on routine clinical data, no a priori power calculation was performed. The analysis should therefore be considered exploratory and hypothesis-generating.

## 3. Results

### 3.1. Study Population

A total of 43 patients treated with the combined rPMS protocol at a private outpatient rehabilitation clinic were included based on available clinical records. Six patients were excluded from the analysis due to incomplete outcome data or failure to complete the full treatment program, resulting in a final analytical sample of 37 patients (23 women and 14 men) with a mean age of 48.63 ± 13.95 years. All patients who completed the full treatment program tolerated the intervention well, and no adverse events were documented in the clinical records during or after therapy.

### 3.2. Clinical Outcomes

**Figure 2:**
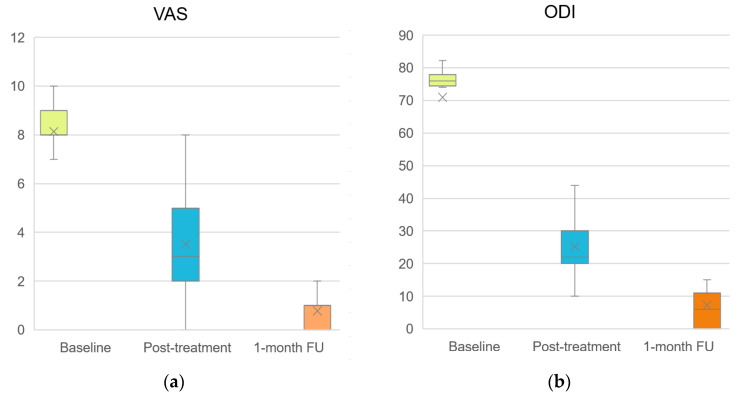
Box plots illustrating the course of (**a**) VAS and (**b**) ODI throughout the treatment period and at the 1-month follow-up. The boxes represent the interquartile range, the horizontal line indicates the median, x represent mean values and whiskers denote the minimum and maximum values.

**Figure 3:**
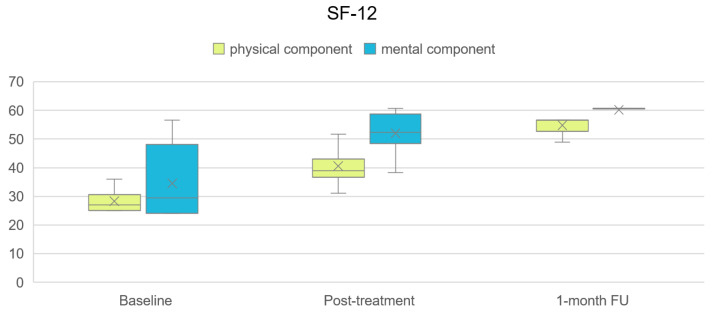
Box plot illustrating the course of SF-12 physical and mental component throughout the treatment period and at the 1-month follow-up. The boxes represent the interquartile range, the horizontal line indicates the median, x represent mean values and whiskers denote the minimum and maximum values.

The baseline values of the assessed parameters, as well as their changes during treatment and at the 1-month follow-up, are presented numerically in Table 1 and graphically illustrated in Figure 2 and Figure 3.

All parameters showed statistically significant within-group changes between baseline and post-treatment, as well as between baseline and the 1-month follow-up (Wilcoxon Signed Rank Test, *p* < 0.001). Improvements observed post-treatment were maintained at follow-up, with further numerical changes recorded across several outcomes.

Analyses of treatment-induced changes, effect sizes, and MCID responder rates are presented in Table 2. Rank-biserial effect sizes approached ±1 across all analyzed intervals, indicating that the vast majority of patients changed in the same direction. MCID responder rates exceeded 90% for most outcomes, and for the SF-12 scores between baseline and one-month follow-up reached 100%.

**Figure 4:**
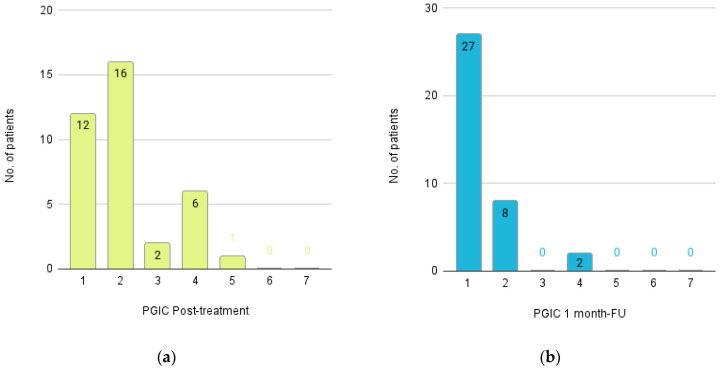
Histogram illustrating Patient Global Impression of Change (PGIC) scores (**a**) immediately after completion of the final treatment session and (**b**) at the 1-month follow-up. PGIC scores range from 1 (very much improved) to 7 (very much worse), with lower scores indicating greater perceived improvement.

The PGIC score improved during the follow-up period from “Much improved” (median: 2; IQR: 1.16) to “Very much improved” (median: 1; IQR: 1). Immediately after completion of the treatment, 92% of patients rated their condition as subjectively improved, increasing to 100% at the 1-month follow-up (Figure 4).

### 3.3. Results of the Literature Review

**Figure 5:**
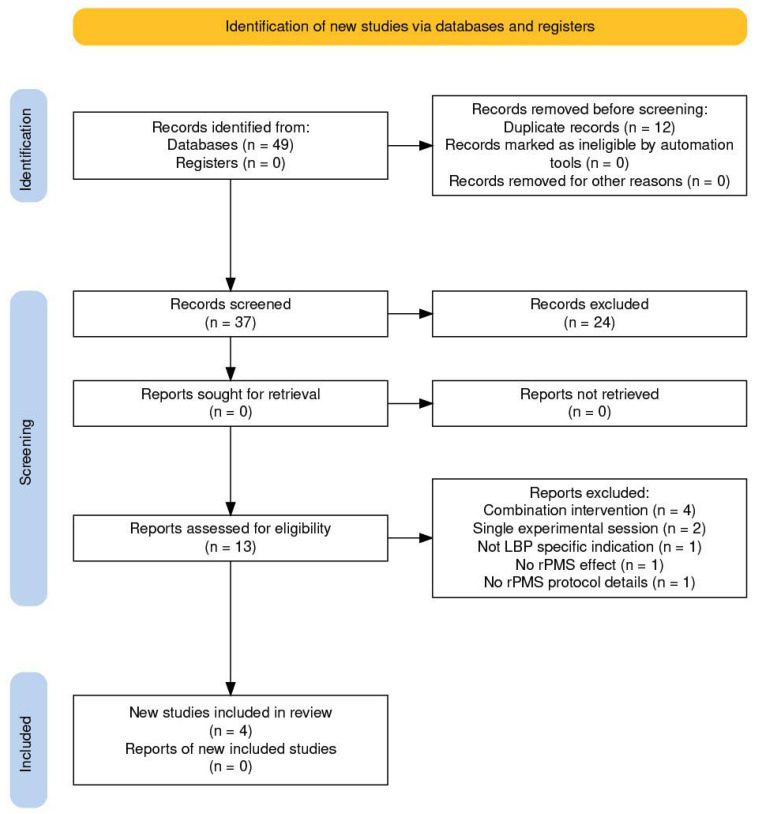
Flow diagram of study identification, screening, eligibility, and inclusion.

A total of 49 records were identified. After removing duplicates and screening titles and abstracts, 13 studies were selected for full-text review. Of these, 4 studies met all inclusion criteria and were included for narrative comparison. The screening process is summarized in the PRISMA flow diagram (Figure 5), and the characteristics of the included studies are presented in Table 3 [34].

## 4. Discussion

This observational case series based on routine clinical data supports the feasibility of a therapy combining static double-coil and dynamic single-coil rPMS for the treatment of LBP within a real-world rehabilitation setting. The findings suggest potential associations with reductions in pain and disability and improvements in quality of life. Importantly, the aim of the present study was to evaluate the combined rPMS protocol as applied in routine clinical practice, rather than to isolate the effects of its individual components.

The clinical relevance of the observed treatment-induced changes was further supported by the calculated effect sizes and MCID responder rates. Rank-biserial effect sizes approached ±1 across all comparisons, indicating that the vast majority of patients changed in the same direction. In addition, more than 95% of patients exceeded the MCID thresholds across all measures between baseline and the one-month follow-up. However, given the single-arm design without a control group, these findings reflect within-group improvement and cannot establish causal treatment efficacy.

Although direct comparison of treatment efficacy across different studies is methodologically limited, the results were contextualized within the existing clinical evidence to provide a pilot-level interpretation of the novel rPMS approach. The present review identified four randomized controlled trials on rPMS for LBP, each with sample sizes ranging from 13 to 15 patients per group [11,35,36,37]. Reported protocols varied considerably, with the number of treatment sessions ranging from 3 to 20 and stimulation times between 5 and 30 min. Importantly, none of these studies included follow-up beyond one week. For example, Jiravichitchai et al. observed an initial improvement after treatment but reported deterioration of outcomes within seven days. Such heterogeneity, together with the absence of longer-term evaluation, makes it difficult to draw consistent conclusions regarding rPMS protocols in LBP. In addition, many potentially relevant studies had to be excluded from the review due to co-interventions such as exercise or injections, which would have confounded the isolated effects of rPMS. Overall, the current body of evidence is characterized by small scale, methodological diversity, and a historical focus on neurological rather than musculoskeletal applications.

Against this background, the present case series provides preliminary real-world data on a combined stimulation protocol not previously described in musculoskeletal LBP. The inclusion of 37 patients offers feasibility insight within routine practice. In contrast to earlier work, which terminated follow-up within days, we extended observation to one month with maintained improvements in pain, disability, and quality of life. Furthermore, the applied protocol—combining a static dual approach with dynamic manual stimulation and extending treatment to both the lumbar spine and lower limbs—represents a novel approach that addresses both axial and peripheral contributors to pain. While numerical reductions in pain and disability appeared greater than those reported in some prior small RCTs, such cross-study comparisons are inherently limited and must be interpreted cautiously due to differences in study design, baseline severity, treatment parameters, and outcome reporting. The observed progression of improvement at follow-up may reflect continued neuromuscular adaptation; however, alternative explanations such as regression to the mean, natural fluctuation of chronic pain, patient expectation, or contextual therapeutic effects cannot be excluded.

The clinical rationale for including lower limb stimulation is supported by previous work in other treatment modalities. Although Forbes did not confirm additional benefit from combining lumbar and subtalar manipulation, more recent findings by Cai et al. and Krawczyk-Wasielewska et al. showed that treatment protocols involving the lower extremities may provide additional improvements in pain relief and functional capacity [38,39,40]. These results are consistent with the concept of convergent innervation and overlapping pain pathways and suggest that combined stimulation of the lumbar spine and lower limbs may be relevant in the management of LBP [41].

This study should be interpreted in light of its limitations. The most significant limitation lies in its single-arm observational design, which precludes direct comparison with either sham stimulation or standard static single-coil rPMS. The reliance on self-reported outcomes and partially remote follow-up assessments introduces the possibility of response bias. Because the analysis was based on routine clinical data, the sample size and study structure were determined by practice conditions rather than prospective trial design. No formal blinding or randomization was implemented. Consequently, improvements observed in this study may partly reflect non-specific treatment effects, including placebo responses, regression to the mean, or contextual factors associated with rehabilitation settings.

While the one-month follow-up provided valuable insight into short-term treatment maintenance, future prospective studies with control groups and longer follow-up durations (≥3–6 months) are warranted to determine the durability and comparative effectiveness of the combined rPMS protocol. In addition, the use of complete-case analysis, including only patients who completed the full treatment protocol and had complete outcome data, may have introduced attrition bias and influenced the observed results. The study population was also heterogeneous, including both acute and chronic LBP cases and patients with varying symptom presentations, without stratification according to clinical subtype, symptom duration, or prior treatment history. Furthermore, due to the composite nature of the intervention, it is not possible to determine the relative contribution of individual treatment components.

In addition to addressing the aforementioned limitations, future research should also focus on evaluating treatment effects in relation to the clinical presentation of LBP. Specifically, it would be valuable to investigate whether the combined protocol demonstrates differential effects in patients presenting with radicular symptoms, or whether comparable benefits are achieved in individuals with chronic versus acute LBP. Given the multifactorial nature of LBP, identifying the precise origin of symptoms is often challenging. Therefore, it would be clinically relevant to explore whether the proposed combined stimulation protocol is universally applicable across different patient subgroups, or whether certain phenotypes may benefit more from conventional, static single-coil stimulation. Such subgroup analyses could help tailor therapeutic strategies and improve individual treatment outcomes. Identifying subgroups that may preferentially benefit from dynamic multi-region stimulation versus conventional static approaches would be clinically relevant.

Despite these limitations, this analysis provides preliminary clinical data on a combined rPMS protocol in musculoskeletal LBP. The findings support the feasibility of this approach and justify further investigation in adequately powered randomized controlled trials.

## 5. Conclusions

The present observational case series based on routine clinical data evaluated a therapy combining static double-coil and dynamic single-coil rPMS and supports the feasibility of this approach for the treatment of LBP. These findings should be considered preliminary and interpreted in light of the study’s observational design and associated methodological and ethical considerations. The observed within-group improvements in pain, disability, and quality of life warrant further investigation under controlled conditions. However, further studies incorporating a control group and extended follow-up periods are essential to verify the potential benefits of simultaneous stimulation of both the lower back and lower limbs in the management of LBP.

## Figures and Tables

**Table 1:** Summary of outcome measures obtained across participants during the treatment course.

		Baseline	Post-Treatment	1-Month FU
VAS	Median (IQR)	8 (1)	3 (3)	1 (1)
	%Δ	n/a	62.5	87.5
ODI	Median (IQR)	76 (3)	22 (10)	6 (11)
	%Δ	n/a	86.84	92.11
SF-12 PCS	Median (IQR)	27.1 (5.57)	39.03 (5.51)	56.58 (3.22)
	%Δ	n/a	56.58	108.78
SF-12 MCS	Median (IQR)	29.52 (10.52)	52.37 (5.76)	60.76 (0)
	%Δ	n/a	60.76	105.83
PGIC	Median (IQR)	n/a	2 (2)	1 (1)

FU: Follow-up; ODI: Oswestry Disability Index; PGIC: Patient Global Impression of Change; SF-12 PCS = Short Form-12 Physical Component Summary; SF-12 MCS = Short Form-12 Mental Component Summary; VAS: Visual Analog Scale; n/a = not available; %Δ = the mean percentage change from baseline across participants.

**Table 2:** Summary of within-subject changes, effect sizes, and MCID responder rates. Values represent median change (Δ) with interquartile range (IQR) in parentheses and Hodges–Lehmann 95% confidence intervals (CI) in brackets. Effect sizes are expressed as rank-biserial correlation (r), with values of approximately 0.1, 0.3, and ≥0.5 interpreted as small, moderate, and large effects, respectively. Values approaching ±1 indicate that most patients changed in the same direction. The MCID (minimal clinically important difference) threshold and the proportion of patients exceeding MCID (% ≥ MCID) are provided to illustrate clinical relevance. Negative Δ values indicate improvement for pain and disability scores (VAS, ODI), while positive Δ values indicate improvement for quality-of-life scores (SF-12 PCS and MCS).

		Median Δ (IQR) [HL 95% CI]	Effect Size	MCID	% ≥ MCID
VAS	Post-Baseline	−5 (−6; −3) [−5.5; −4.5]	−1.00	≥2 points	94.59%
	%Δ	n/a	62.5		
	FU-Post	−2 (−4; −1) [−3.5; −2.0]	−1.00		70.27%
	FU-Baseline	−8 (−9; −7) [−8.0; −7.0]	−1.00		97.3%
ODI	Post-Baseline	−49 (−59; −17) [−52.5; −40.0]	−1.00	≥10 points	94.59%
	%Δ	n/a	86.84		
	FU-Post	−17 (−22; −15) [−22.0; −15.0]	−0.96		89.19%
	FU-Baseline	−70 (−83; −33) [−72.0; −58.0]	−1.00		97.3%
SF-12 PCS	Post-Baseline	12.46 (9.58; 18.00) [10.29; 14.64]	1.00	≥3.4 points	89.19%
	FU-Post	15.98 (12.80; 17.55) [13.08; 17.29]	0.99		91.89%
	FU-Baseline	27.27 (23.96; 30.01) [25.62; 28.37]	1.00		100%
SF-12 MCS	Post-Baseline	18.17 (14.24; 24.23) [14.24; 21.69]	0.97	≥4 points	81.08%
	FU-Post	8.39 (4.18; 11.81) [6.42; 10.45]	1.00		75.68%
	FU-Baseline	28.41 (25.22; 36.14) [21.80; 31.87]	1.00		100%

FU = follow-up; HL = Hodges–Lehmann; MCS = Short Form-12 Mental Component Summary; ODI = Oswestry Disability Index; PCS = Short Form-12 Physical Component Summary; VAS = Visual Analog Scale; n/a = not available.

**Table 3:** Summary of studies identified in the literature search, including study design, patient population, intervention protocols, and reported outcomes.

Study	Design	Indication	n	Protocol	Outcome	Results
Tammasse et al. 2021 [35]	RCT	CLBP	30 (15/15)	5 × 30 min	ISI, NPRS	ISI: 33.6% vs. 7% NPRS: 29.7% vs. 20.3%
Kim & Nam 2021 [11]	RCT	CLBP	30 (15/15)	20 × 10 min	VAS, KODI, SF-36	VAS: 58.3% vs. 12.5% KODI: 44.4% vs. 2.7% SF-36: −0.35% vs. −1.32%
Jiravichitchai 2024 [36]	RCT	CLBP	30 (15/15)	3 × 15 min	VAS, ODI, RMDQ	VAS (post): 52.1% vs. 25.0% VAS (FU): 45.8% vs. 25.0% ODI (FU): 43.2% vs. 14.7% RMDQ (FU): 3.0 pt vs. 2.0 pt
Lim et al. 2018 [37]	Pilot RCT	Acute LBP	26 (13/13)	10 × 20 min	VAS, ODI, RMDQ	VAS: 57.5% vs. 42.17% ODI: 55.4% vs. 29.25% RMDQ: 65.55% vs. 44.09%

CLBP: Chronic Low Back Pain; ISI: Insomnia Severity Index; KODI: Korean Oswestry Disability Index; LBP: Low Back Pain; NPRS: Numeric Pain Rating Scale; ODI: Oswestry Disability Index; RCT: Randomized Controlled Trial; RMDQ: Rolland Morris Disability Questionnaire; VAS: Visual Analog Scale.

## Data Availability

The data presented in this study are available on request from the corresponding author due to data are not publicly available due to privacy or ethical restrictions.

## Abbreviations

The following abbreviations are used in this manuscript:

rPMS	Repetitive peripheral magnetic stimulation
LBP	Low back pain
MCID	Minimal clinically important difference
TMS	Transcranial magnetic stimulation
VAS	Visual analog scale
ODI	Oswestry disability index
PGIC	Patient Global Impression of Change
SF-12	Short Form-12 Health Survey
PCS	Physical component summary
MCS	Mental component summary
IQR	Interquartile range
CI	Confidence interval
